# Brain Function in Gulf War Illness (GWI) and Associated Mental Health Comorbidities

**Published:** 2018-07-19

**Authors:** Brian E. Engdahl, Lisa M. James, Ryan D. Miller, Arthur C. Leuthold, Scott M. Lewis, Adam F. Carpenter, Apostolos P. Georgopoulos

**Affiliations:** 1 Brain Sciences Center, Department of Veterans Affairs Health Care System, Minneapolis, Minnesota, USA; 2 Department of Neuroscience, University of Minnesota Medical School, Minneapolis, Minnesota, USA; 3 Department of Psychology, University of Minnesota, Minneapolis, Minnesota, USA; 4 Center for Cognitive Sciences, University of Minnesota, Minneapolis, Minnesota, USA; 5 Department of Psychiatry, University of Minnesota Medical School, Minneapolis, Minnesota, USA; 6 Department of Neurology, University of Minnesota Medical School, Minneapolis, Minnesota, USA

**Keywords:** Gulf War Illness (GWI), Posttraumatic Stress Disorder (PTSD), Magnetoencephalography, Veterans

## Abstract

GWI has affected a substantial number of Gulf War (GW) veterans. The disease involves several organ systems among which the brain is most prominent. Neurological, cognitive and mood-related (NCM) symptoms frequently dominate and are at the root of chronic ill-health and disability in veterans suffering from GWI. In addition, such symptoms frequently co-occur with diagnosable mental health disorders, predominantly posttraumatic stress disorder (PTSD). Here we investigated the possibility that increased GWI severity leads, above a threshold, to a diagnosable mental health disorder (excluding psychosis). For this purpose, we used, in separate analyses, symptom severity scores and resting-state brain functional connectivity patterns, as determined by magnetoencephalography (MEG). Two-hundred-thirty GW-era veterans participated in this study. They completed diagnostic interviews to establish the presence of GWI and assess mental health status. This distinguished 3 groups: healthy controls (N = 41), veterans with GWI and no mental illness (GWI group, N = 91), and veterans with both GWI and mental health disorder (GWI+MH, N = 98). For each veteran, symptom severity scores in the 6 GWI domains (fatigue, pain, NCM, skin, gastrointestinal, respiratory) were available as well as 9 summary measures of the distribution of Synchronous Neural Interactions (SNI) derived from the MEG recordings. We tested the hypothesis that, in the presence of GWI, the appearance of a diagnosable mental health disorder may depend on GWI symptom severity. For that purpose, we performed a logistic regression on the GWI population, where the presence (or absence) of the MH disorder was the dependent variable and the age- and gender-adjusted GWI severity in the 6-symptom domains were the predictors. The outcome was the probability that a participant will have MH disorder or not. Similarly, we tested the hypothesis that the presence of the MH disorder can be predicted by the SNI distribution patterns by performing a second logistic regression as above but with the 9 SNI measures as predictors. We found GWI symptom severity differed significantly across groups (GWI+MH > GWI > Control). SNI distributions of the GWI group also differed significantly from the other groups in a systematic hemispheric pattern, such that the presence of GWI involved predominantly the left hemisphere, and presence of mental health disorders involved, in addition, the right hemisphere. Both logistic regressions yielded highly significant outcomes, demonstrating that both GWI symptom severity and SNI distribution measures can predict the presence of MH disorder in GWI. Remarkably, the prediction probabilities for MH presence derived from the symptom-based and SNI-based logistic regressions were positively and highly statistically significantly correlated. Taken together, both objective (neural) and subjective (symptoms) indices suggest that GWI is distinct from healthy controls and varies in severity in a continuum that leads, at the higher end, to a diagnosable MH disorder. The positive correlation between the GWI symptom-based and brain-based predicted classifications provides a key link between GWI symptom severity and synchronous neural interactions in the context of mental illness.

## Introduction

Many Allied military personnel who served in the 1990–1991 Persian Gulf War experience various chronic physical and neurocognitive complaints, now commonly referred to as Gulf War Illness (GWI). At least 25% of Gulf War veterans have been affected by diffuse symptoms such as fatigue, musculoskeletal pain, neurological/cognitive/mood (NCM) complaints, respiratory symptoms, gastrointestinal problems, and rashes^1–4^. Several population-based studies have demonstrated that these symptoms occur at significantly higher rates in Gulf War veterans relative to their non-deployed peers and other veteran groups^1,3,4^. However, these symptoms typically do not meet criteria for established medical diagnoses, fueling speculation that they primarily reflect psychological distress and related psychiatric disorders (e.g., Posttraumatic Stress Disorder, PTSD) superimposed on vulnerable organ systems^5,6^. This has been refuted^7^ but it remains the case that the physical and NCM symptoms most commonly found in Gulf War veterans are primarily based on self-report. There are currently no definitive biological indicators of GWI.

Brain-based objective indicators of psychiatric and medical disorders are increasingly being identified, allowing a fresh approach to this challenge. Burgeoning evidence supports the utility of magnetoencephalography (MEG) in identifying aberrant and disease-specific neural activity^8–11^. One approach focuses on characteristic anomalies in synchronous neural interactions (SNI) derived from task-free MEG. Using that approach, we have demonstrated that SNI anomalies accurately discriminate various brain diseases including PTSD, multiple sclerosis, Alzheimer’s disease, schizophrenia, Sjögren’s syndrome, temporomandibular pain disorder, and chronic alcoholism from normal healthy brain function^8^, providing compelling evidence of candidate biomarkers.

Similarly, we hypothesized that GWI could also be characterized by SNI abnormalities, i.e., neural miscommunication patterns. Indeed, in previous studies, we documented such abnormalities in comparison to healthy controls^12^, in relation to the protective role exerted by 6 alleles of the Human Leukocyte Antigen^13,14^, and in relation to known autoimmune disorders^15^. Here, we examine the extent to which SNI anomalies enable discrimination among veterans with GWI, GWI plus mental health (MH) disorders, and healthy control veterans. We also sought to evaluate and compare the GWI symptom profile in each of these groups, hypothesizing that subjective (i.e., self-reported) differences would parallel brain-based objective differences. Finally, we tested the hypothesis that increased GWI severity may lead, above a threshold, to a diagnosable mental health disorder. For this purpose, we used, in separate analyses, symptom severity scores and resting-state brain functional connectivity patterns, as determined by SNI, to predict whether participants suffering from GWI would also have a diagnosable mental health disorder.

## Materials and Methods

### Study participants

A total of 230 Gulf War veterans participated in this study as paid volunteers. VA medical records were reviewed to identify potential participants. Veterans who had completed the Gulf War Registry Examination^16^ and did not meet exclusionary criteria were recruited for participation. Exclusionary criteria included cardiac pacemakers or embedded ferrous metal (due to magnetic effects on MEG), central nervous system disorders (e.g. Parkinson’s disease, dementia, cerebral vascular accidents, a history of traumatic brain injury, etc.), lifetime psychotic diagnoses, and current alcohol or drug dependence. Veterans who might have difficulty with the protocol or were taking medications that might interfere with brain scan results were also excluded. Study participants provided written informed consent prior to initiating study procedures and participants were compensated for their time. The study protocol was approved by the Institutional Review Board at the Minneapolis VA Health Care System.

All participants (Table 1) completed diagnostic interviews and underwent a MEG scan. The Clinician-Administered PTSD Scale for *DSM-IV* (CAPS)^17^ and the PTSD Checklist (PCL)^18^ were used to evaluate current PTSD diagnostic status. Non-PTSD Axis I diagnostic status was determined using the Structured Clinical Interview for DSM-IV-TR Axis I Disorders (SCID)^19^. SCID screening questions were administered to all participants; positive screening items were further evaluated with the relevant SCID module. All participants completed a GWI symptom questionnaire developed for use in Kansas Gulf War veterans^4^ that evaluates the presence and severity of 6 kinds of symptoms characteristic of GWI – namely, fatigue, pain, neurological/mood/cognitive, gastrointestinal, skin rashes, and respiratory. Items are rated on a scale from 0 to 3 (absent, mild, moderate and severe, respectively). The questionnaire permits determination of case status according to either the Centers for Disease Control and Prevention (CDC) criteria^1^ or the Kansas GWI case definition^4^. Participants meeting either set of criteria were included in the analyses. Participants were classified into the following groups based on symptom presentation: healthy controls (N = 41), GWI (N = 91), and GWI+MH (N = 98). Of those with co-occurring mental health conditions, 64% met criteria for current (including subthreshold) PTSD, 57% met criteria for a mood disorder, 11% met criteria for an anxiety disorder other than PTSD, and 17% met criteria for some other diagnosis (e.g. adjustment disorder, eating disorder). Finally, none of the controls were receiving any psychotropic medication. Of the participants suffering from GWI, 24/91 participants from the GWI group and 60/98 from the GWI+MH group were under medication; the data from the remainder (67 GWI and 38 GWI+MH) were used to check on the results obtained from the full sample.

### Data acquisition

As described previously^8,20^, subjects lay supine within the electromagnetically shielded chamber and fixated their eyes on a spot ~ 65 cm in front of them, for the 60 s. MEG data were acquired using a 248-channel axial gradiometer system (Magnes 3600WH, 4-D Neuroimaging, San Diego, CA), band-filtered between 0.1 and 400 Hz, and sampled at 1017.25 Hz. Data with artifacts (e.g. from non-removable metal or excessive subject motion) were eliminated from further analysis.

### Data analysis

Standard statistical methods were used to analyze symptom data (analysis of covariance [ANCOVA], correlation, etc.)^21^. In addition, multidimensional scaling, factor analysis (without rotation) and logistic regression were performed, as needed, using the IBM-SPSS statistical package (version 23).

Single-trial MEG data from all sensors underwent prewhitening^22,23^ using a (50,1,1) ARIMA model. The Matlab package (version 2011b) was used to fit the model and obtain innovations (i.e. residuals). All possible pairwise zero-lag cross correlations (N = 30,628, given 248 sensors) were computed between the pre-whitened MEG time series. Finally, the partial zero-lag cross correlations $PCC_{ij}^{0}$ between i and j sensors were computed for all sensor pairs (synchronous neural interactions; SNI); thus, for any given pair of sensors (from a total of 248) the effects of the remaining 246 sensors were partialed out. The $PCC_{ij}^{0}$ was transformed to $Z_{ij}^{0}$ using Fisher’s (Fisher, 1958) z-transformation^24^ to normalize its distribution:

(1) $$\text{SNI}=z_{ij}^{0}=\;\text{atanh}{\;}_{(}PCC_{ij)}^{0}$$

Distributions of $Z_{ij}^{0}$ were compared between groups using the Kolmogorov-Smirnov test. This test provided two outcomes. First, it assessed the statistical significance between the two distributions tested in a pair; and second, it yielded a statistic (Smirnov’s Z^25^) which served as proximity (e.g. distance) measure in a subsequent multidimensional scaling analysis (MDS)^26^. A nonmetric MDS analysis was performed using the PROSCAL procedure of the IBM-SPSS statistical package, version 23. The MDS is a well-established dimension-reduction method^27^ that has proved valuable in psychological^26,28^, behavioral^29^, psychiatric^30^, and neuroscientific^31–35^ studies. It typically reveals underlying associations between variables that may be hidden when embedded in a multidimensional space. This analysis indicated that the groups were clearly separated in the two MDS dimensions (see below). To ensure the validity of this finding, we additionally performed a weighted MDS analysis using 100 proximity matrices obtained by calculating Snirnov’s Z statistic in 100 bootstrap samples of the populations, where each such sample consisted of the same number of subjects chosen randomly with replacement from the original populations.

Finally, parametric group differences in $Z_{ij}^{0}$ (SNI) between groups (Control vs. GWI, Control vs. GWI+MH, GWI vs. GWI+MH) were evaluated for each (*i,j*) pair of sensors using an ANCOVA, where age and gender served as covariates. For visualization purposes, we used a nominal threshold of P < 0.01 (uncorrected) to plot SNIs exceeding that threshold. These plots (see below) indicated differential involvements of SNIs within the left hemisphere, the right hemisphere, and between hemispheres. For that reason, we extracted, for each participant, 3 summary statistics of SNIs (mean, standard deviation, and the ratio of mean over its standard error) for each one of these domains (left, right and interhemispheric SNIs) that captured the essential properties of SNI distributions, for a total of 9 SNI-derived measures.

## Results

### Demographics

All groups comprised predominantly men (Table 1) in similar proportions (statistically not significantly different, test of two proportions). The control group participants were significantly older than those e in the GWI+MH group by an average of 3.4 y. All statistical analyses were performed using gender and age as covariates.

### GWI symptom severity across groups

**Figure 1:**
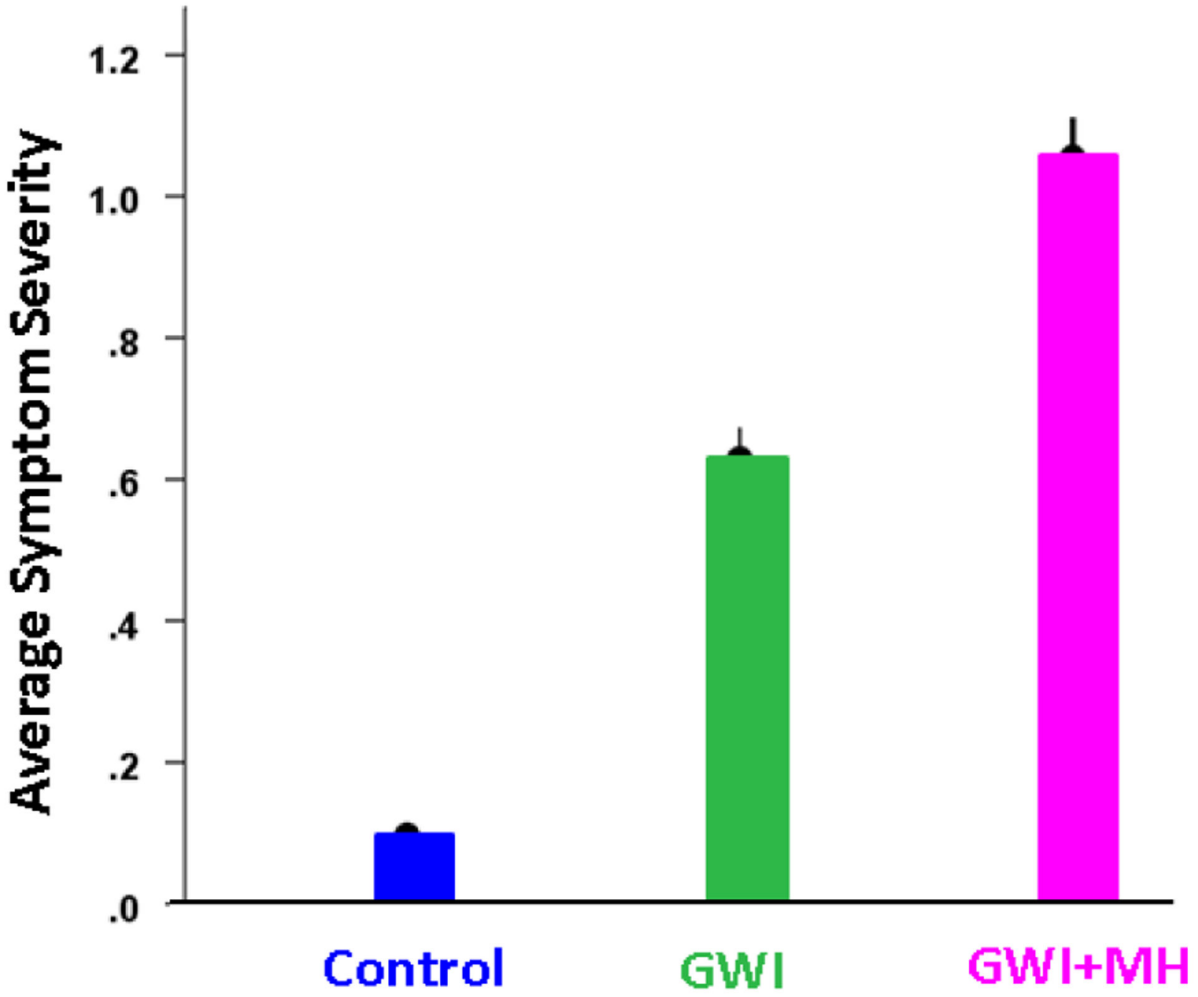
Overall (mean ± SEM) of GWI symptom severity per group.

The average GWI symptom severity within the 6 categories of symptoms (fatigue, pain, neurocognitive problems, skin rashes, gastrointestinal problems, and respiratory problems) is shown in Figure 1. The Group main effect in the ANCOVA (with gender and age as covariates) was highly significant (P < 10^−21^), as were all 3 pairwise comparisons (P < 10^−8^). The average symptom severity in the GWI and GWI+MH groups was 5.6x and 9.3x higher than in the control group, respectively; and that in the GWI+MH group was 1.7x higher than the GWI group. Of note, only 16 (6.9%) of study participants were free of any symptoms. This means that literally “asymptomatic” control subjects are rare within the GW-era veterans, or at least those included in our Gulf War Registry.

**Figure 2:**
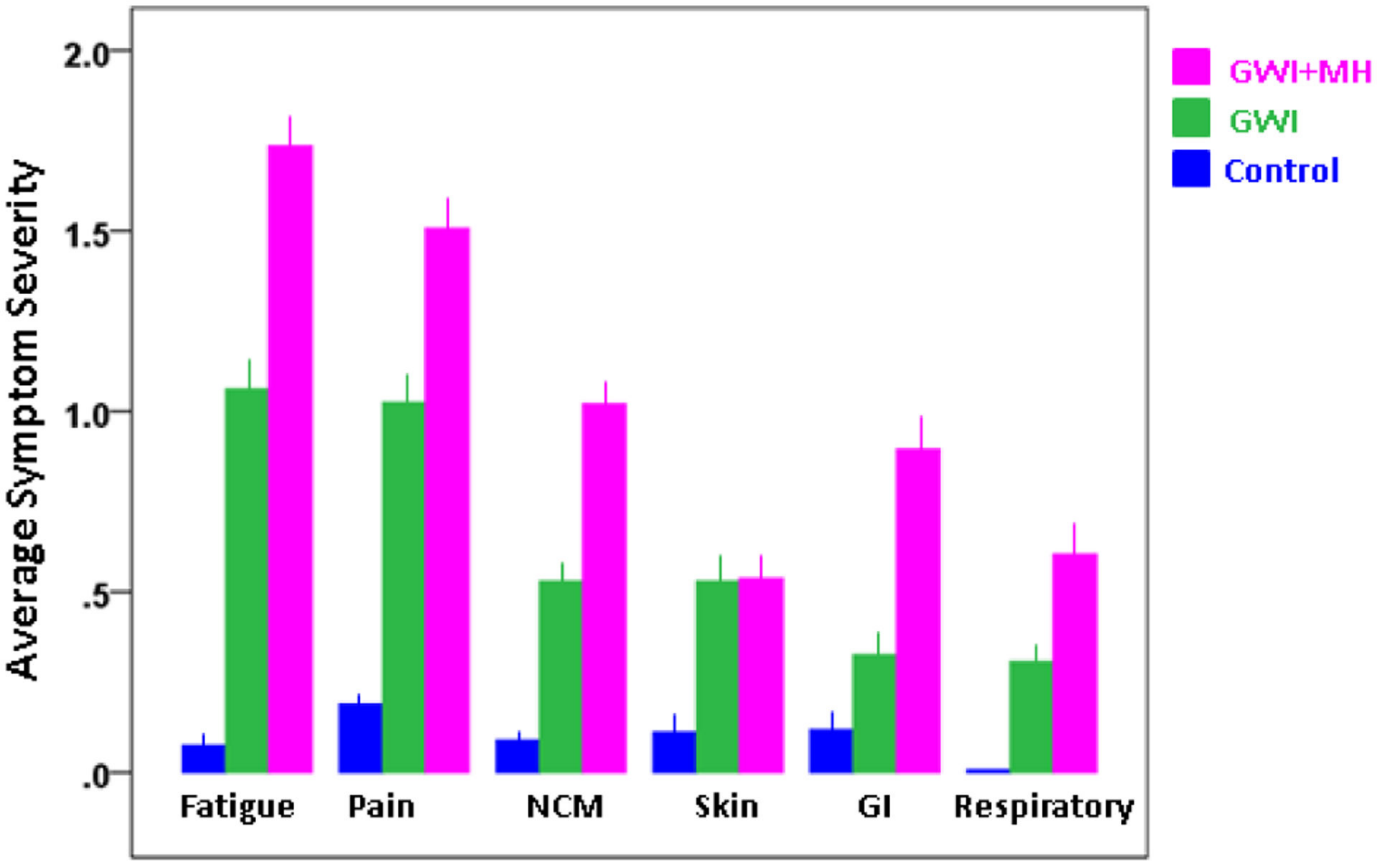
Specific GWI symptom severity (mean ± SEM) per group.

**Figure 3:**
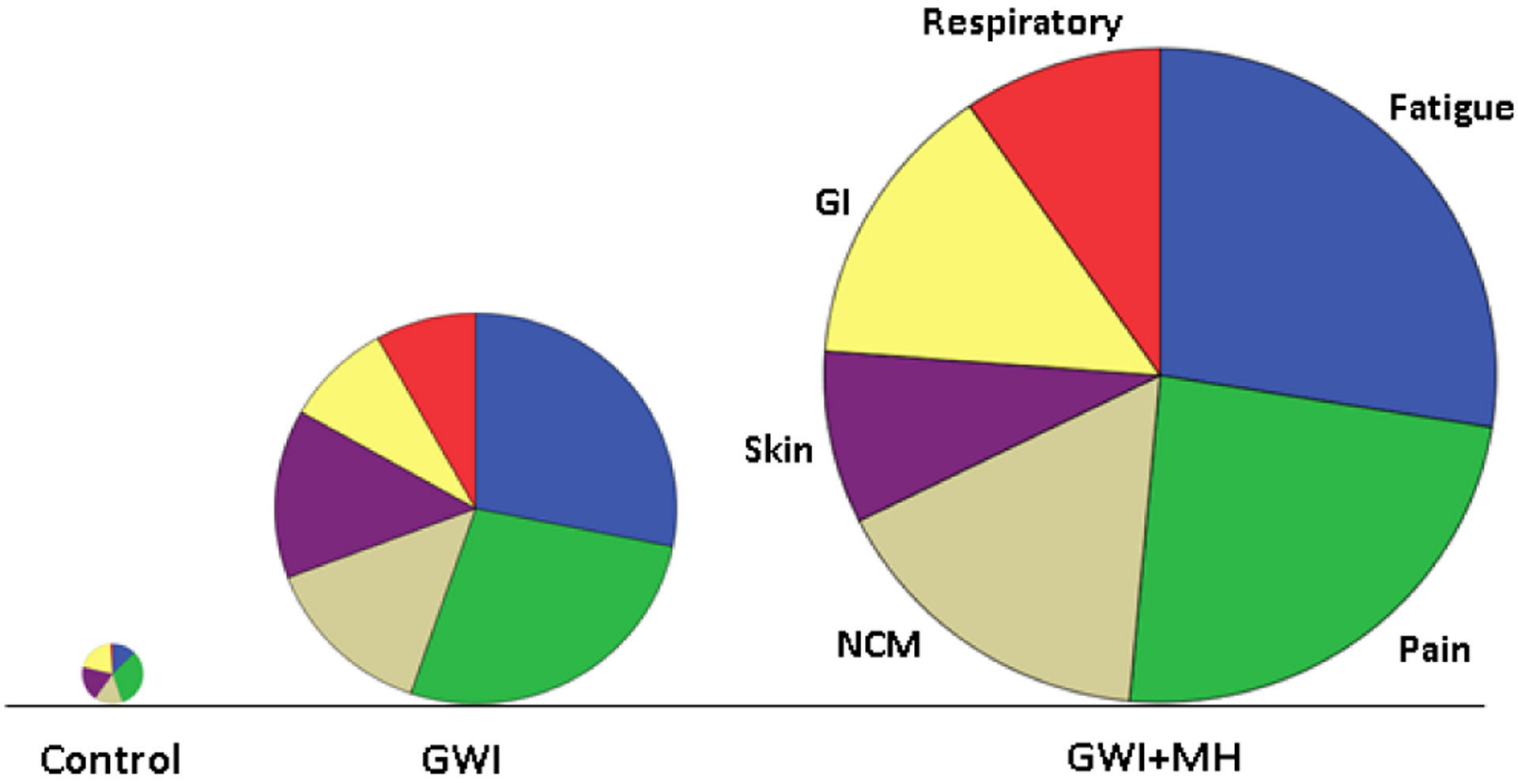
Pie plots of the data in Table 2 where the radius of the pie is proportional to the overall symptom severity. GI, gastrointestinal; NCM, neurological-cognitive-mood.

Symptom severity for specific domains is shown in Figure 2. It can be seen that, for all domains, severity was highest for the GWI+MH group, lower for the GWI group, and lowest for the control group. All pairwise comparisons showed statistically significant differences (P < 0.01, ANCOVA with gender and age as covariates) except for GWI vs. GWI+MH for Skin, and Control vs. GWI for Gastrointestinal, which were not significant. Symptom severity in the Control group was not significantly correlated with that in the GWI group (P = 0.22) or the GWI+MH group (P = 0.26). In contrast, mean symptom severity was highly correlated between GWI and GWI+MH groups (*r* = 0.894, P = 0.016, N = 6 symptom domains). This was true also for the relative distributions of the 6 symptom severity in the 3 groups, as percentages of total symptom severity (Table 2 and Figure 3); the major difference among groups was the average symptom severity (diameter of the pie in Figure 3).

**Figure 4:**
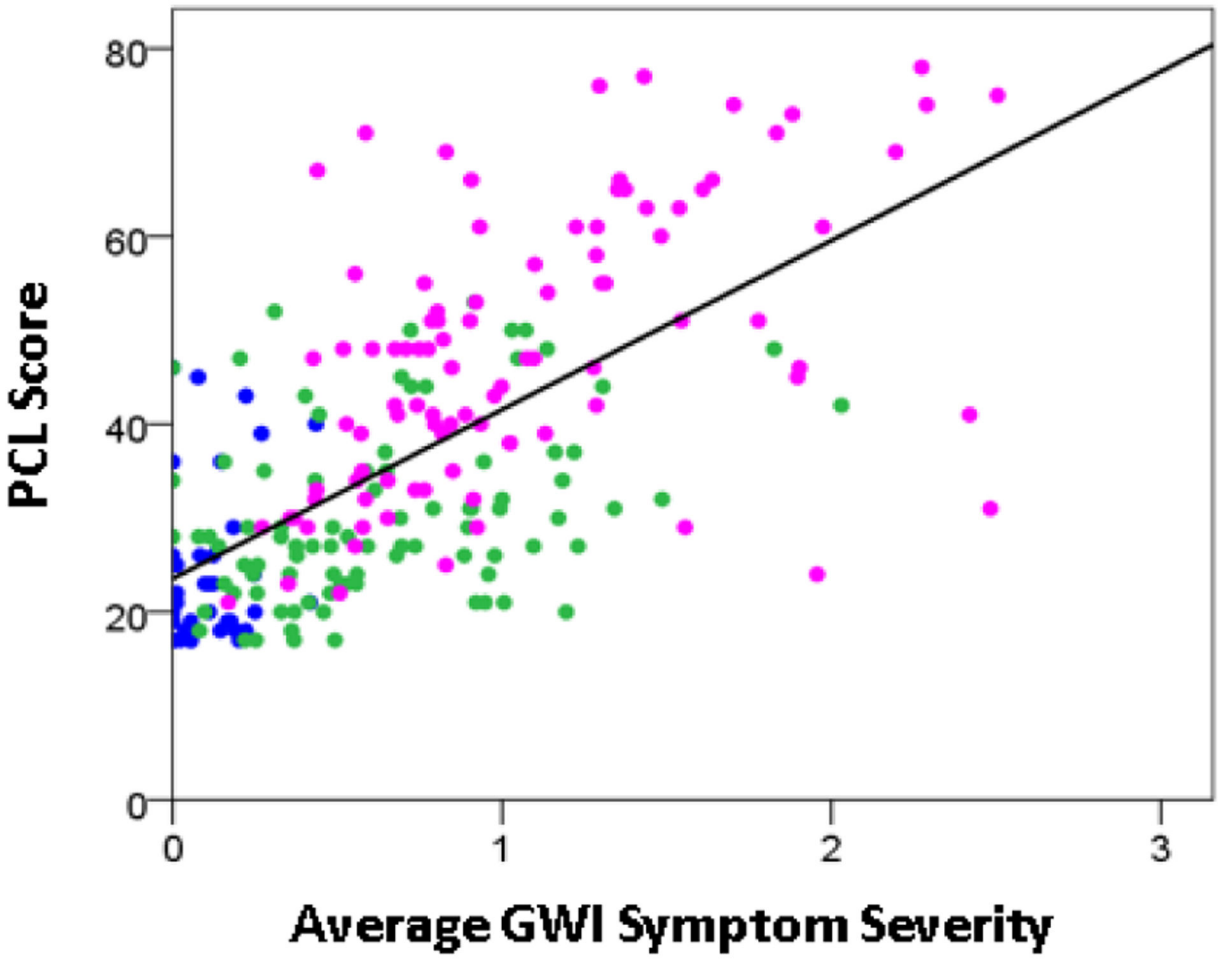
PCL score is plotted against GWI severity score for the Control (blue), GWI (green) and GWI+MH (magenta) groups. (See text for details).

Finally, we assessed the possible association between the PCL score and GWI symptom severity in two ways. First, we performed an ANCOVA where the PCL score was the dependent variable, the Group (Control, GWI, GWI+MH) was a fixed factor, and gender and age were covariates. We found that GWI+MH had significantly higher scores, as compared to Control (P = 5.24 × 10^−23^) and GWI (P = 0.002) groups. And second, we evaluated the dependence of the PCL score on the average GWI symptom severity score across groups by performing a regression analysis. We found that the PCL score increased as a linear function of mean GWI severity score (Figure 4). This dependence was highly statistically significant (P = 1.55 × 10^−29^, t-test on the PCV vs. GWI regression coefficient, R^2^ = 0.429, N = 230).

### Factor analysis of GWI symptom severity across groups

**Figure 5:**
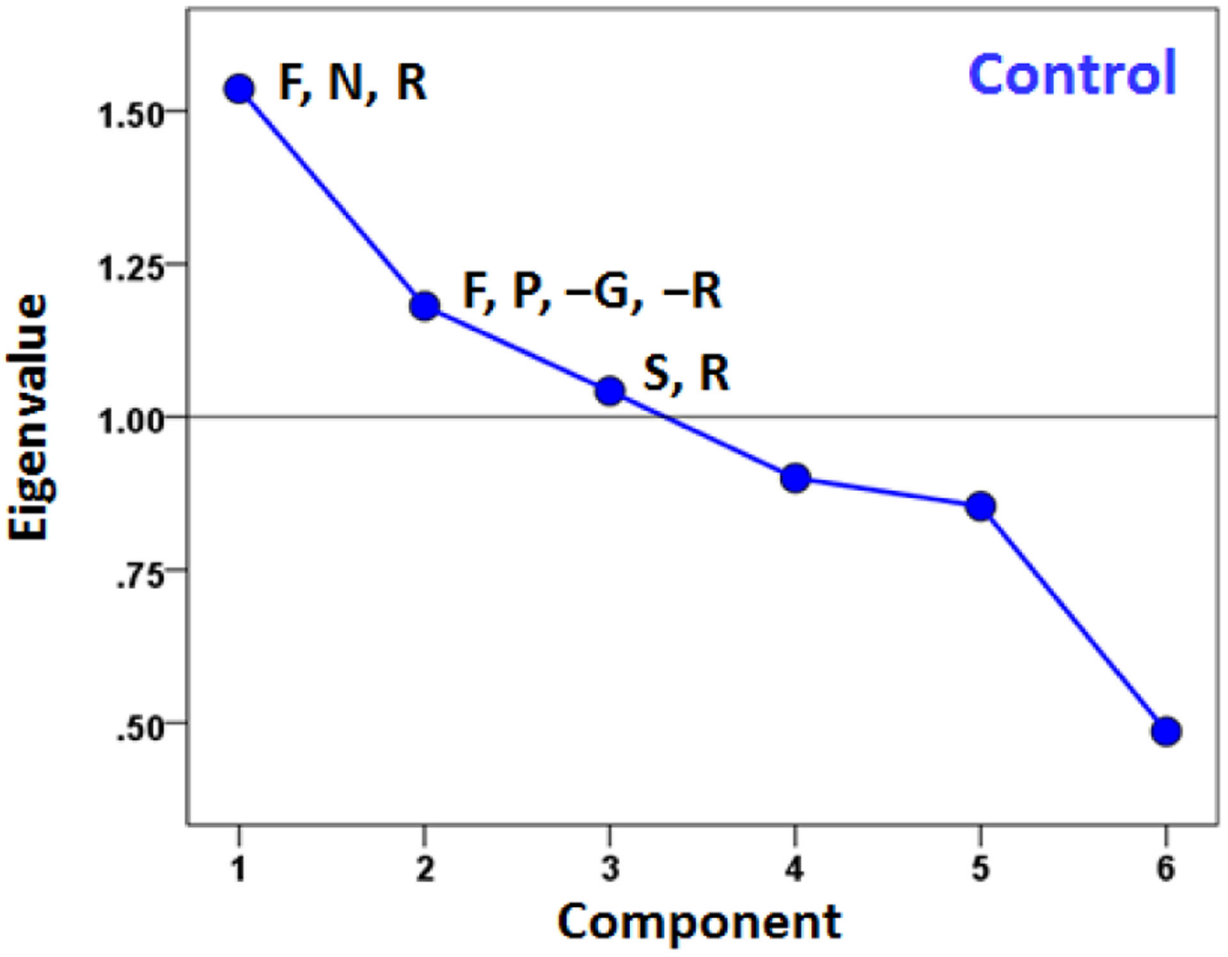
Scree plot from factor analysis of symptom severity in the Control group. F, fatigue; P, pain; N, neurocognitive-mood; S, skin; G, gastrointestinal; R, respiratory. Signs (positive, negative) indicate the sign of loading of a specific symptom onto the corresponding component with absolute value of component score > 0.4.

**Figure 6:**
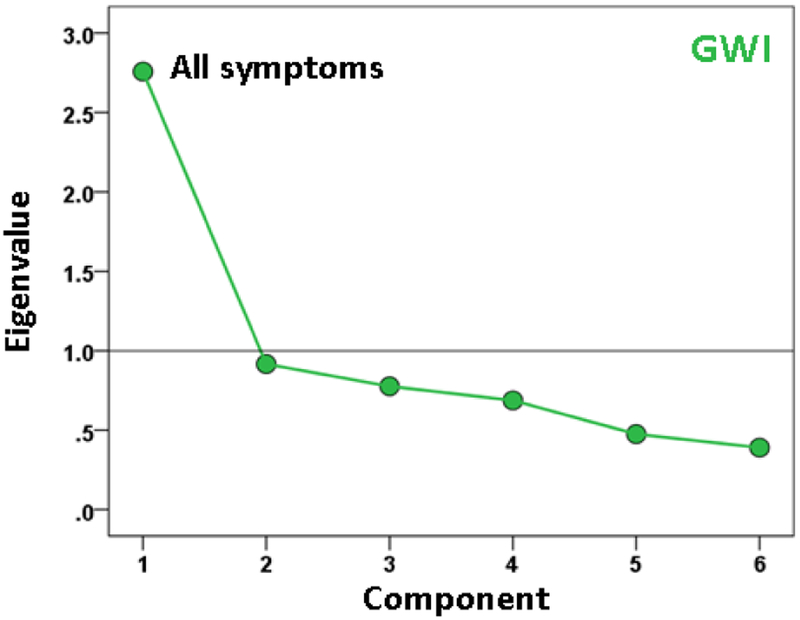
Scree plot from factor analysis of symptom severity in the GWI group.

**Figure 7:**
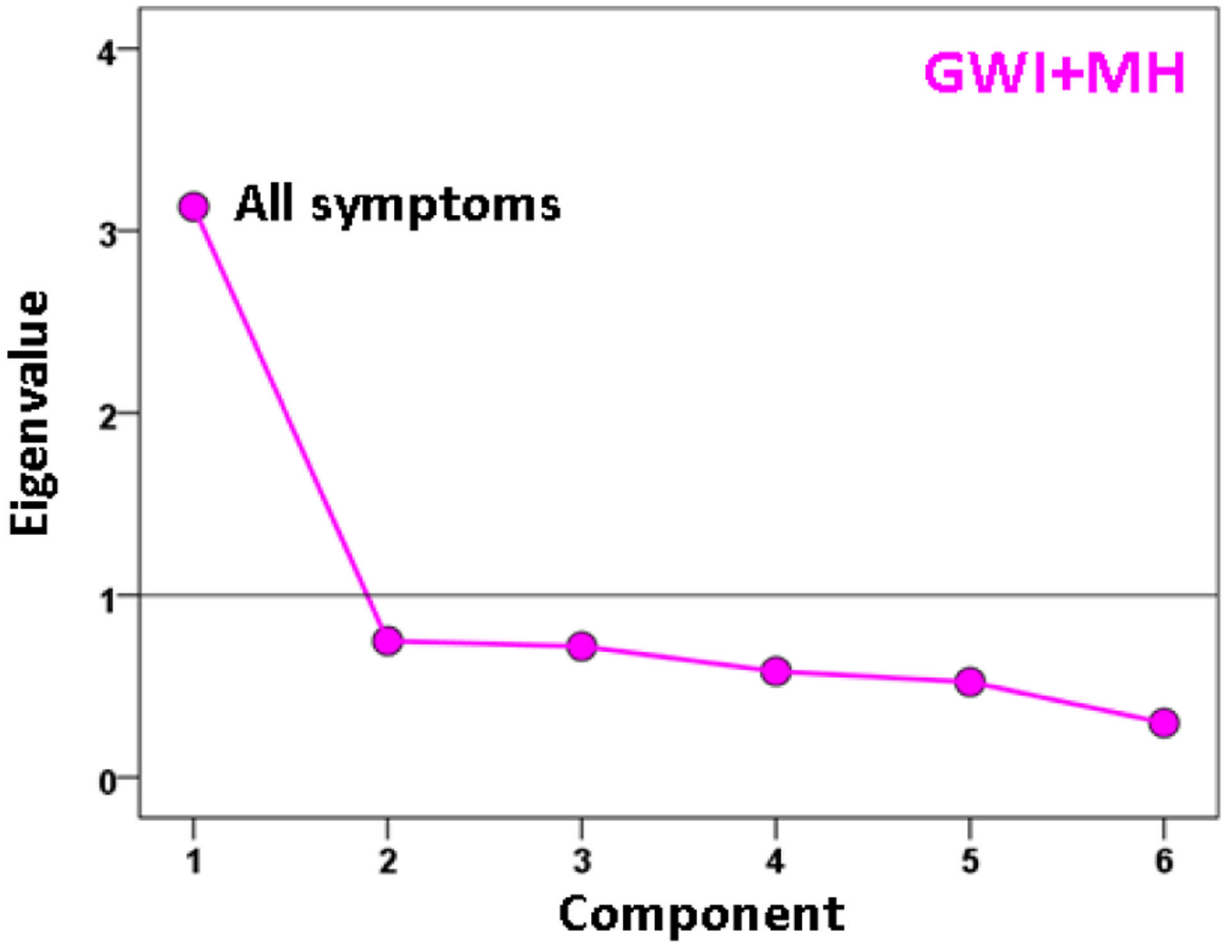
Scree plot from factor analysis of symptom severity in the GWI+MH group.

A different question concerns the internal structure of symptom severity, namely to what extent subsets of them may represent different latent variables (component factors), and how such variables may differ across groups. We explored this idea by performing a factor analysis of symptom severity scores within each group. The results are shown in the scree plots of Figures. 5–7. For the Control group (Fig. 5), three components were extracted (eigenvalues > 1), which suggests a diversified symptom correlation structure. In contrast, a single, major component comprising all symptoms were extracted for both GWI (Fig. 6) and GWI+MH (Fig. 7) groups. This indicates a commonality among the various symptoms shared by both groups.

### Development of a mental health disorder with increasing GWI symptom severity

**Figure 8:**
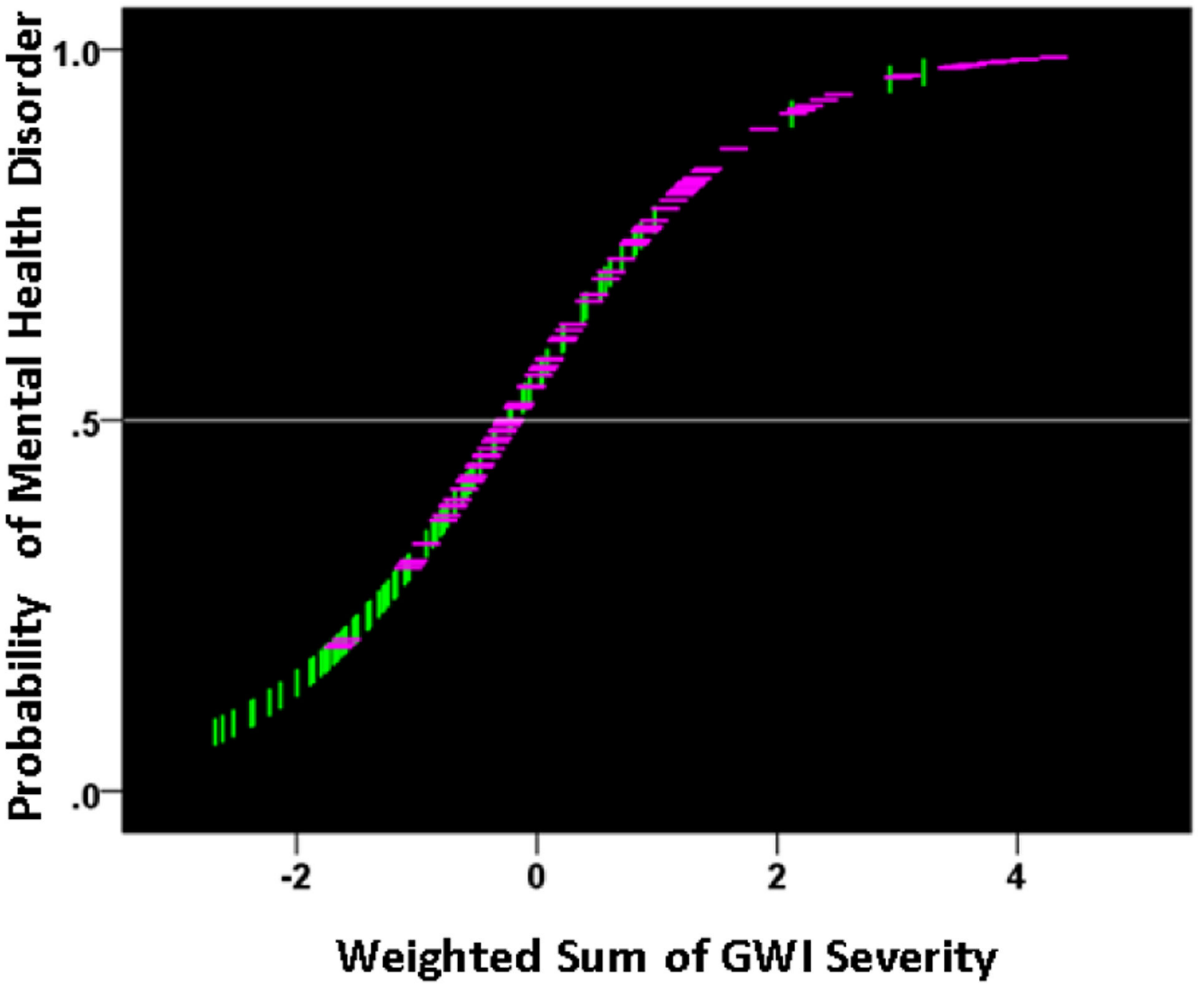
The probability of presence of mental health disorder is plotted against the weighted sum of the 6 GWI severity, where the weights are the coefficients provided by the logistic regression. (see text for details.) Vertical green lines indicate participants without mental health problems; horizontal magenta lines indicate participants with mental health problems.

The findings above that GWI symptom severity is higher in the presence of a mental health disorder suggest, conversely, that increasing GWI symptom severity (a proxy for GWI severity overall) may actually lead to the development of a diagnosable mental health disorder. We investigated this idea by performing a binary logistic regression analysis in the combined GWI and GWI+MH populations (i.e. excluding controls). We used only data for which all participants had valid (i.e. non-missing) severity scores for all 6 GWI symptoms (N = 91 GWI and N = 83 GWI+MH participants, total N = 174). In this analysis, the presence (or absence) of mental illness was the dependent variable and the severity of the 6 GWI symptoms (adjusted for age and gender) were the independent variables. (These adjusted values were obtained by performing a multiple linear regression of each symptom on age and gender, and using the residuals as the independent variables in the logistic regression.) There are two outcomes of interest from this analysis, namely (a) the probability that an individual has or does not have a mental health disorder, and (b) the assignment of an individual to one of the two groups (based on a 0.5 probability threshold), resulting in a two-way classification table. The analysis also provides derived coefficients for each one of the 6 symptoms which, used as weights for the corresponding symptom variables, yield a linearly weighted sum that is, actually, the effective independent variable in the analysis. (This sum can take negative and positive values, depending on the weights.) We found a highly significant dependence of the presence of mental health illness on the GWI symptom severity. Figure 8 plots the probability of a particular individual to have mental illness against the weighted sum of GWI symptom severity. It can be seen that this probability increases as a sigmoid (logistic) function of GWI symptom severity. The Nagelkerke R^2^ (a measure ranging from zero to 1, analogous to the R^2^ in linear regression) was 0.348.

**Figure 9:**
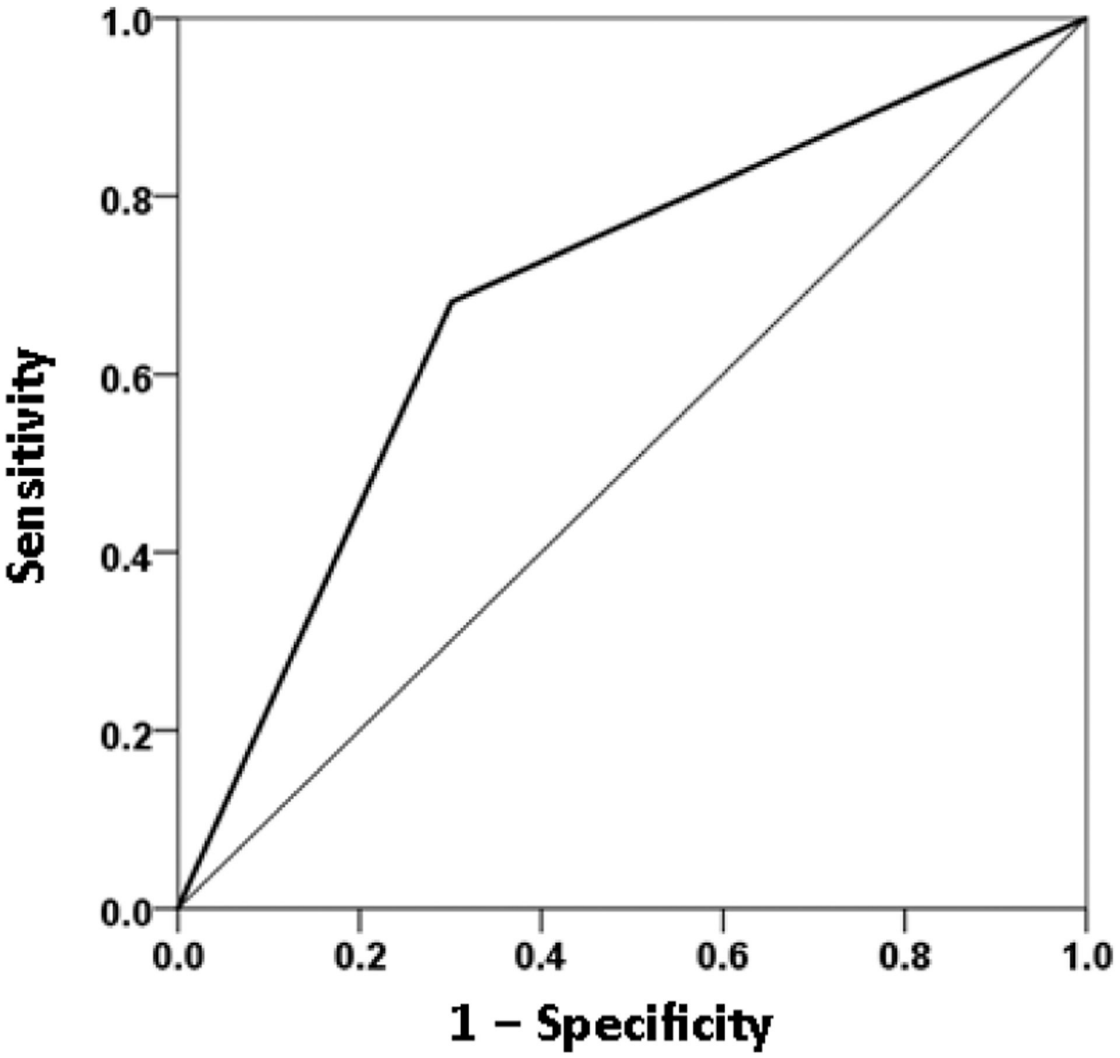
ROC curve yielded by the logistic regression of presence/absence of mental health disorder against the severity of the 6 GWI symptoms, adjusted for age and gender. (See text for details.)

The overall classification rate (with probability = 0.5 as the group discrimination border) was 69% (69.9% specificity, 68.1% sensitivity). The following statistics derived from the two-way table documented the high statistical significance of the obtained classification: χ^2^ = 25.1 (P < 10^−6^, 2-sided); probability of Fisher’s exact test < 10^−6^, 2-sided; odds ratio = 4.96 (asymptotic 2-sided significance P = 0.000001), lower bound of 95% confidence interval (CI) = 2.61, upper bound = 9.44). The receiver operating characteristic (ROC) curve (Figure 9) was 0.69, its asymptotic standard error 0.041, the asymptotic significance 0.000015, the lower bound of 95% CI 0.61 and the upper bound 0.77.

### Group Differences in neural functioning

**Figure 10:**
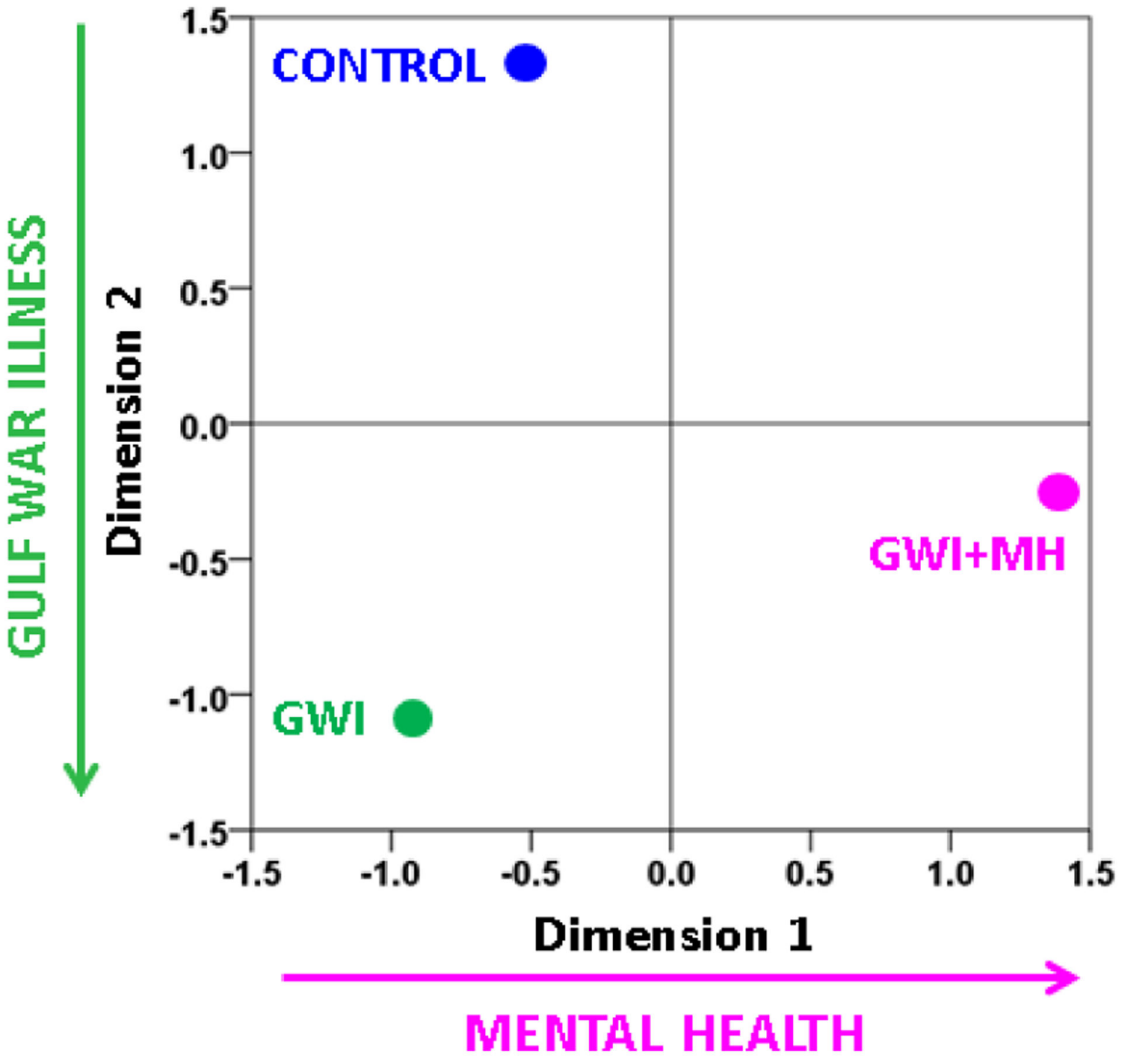
Derived Group configuration from the weighted MDS analysis of 100 bootstraps. (Normalized Raw Stress = 0.00025, Dispersion Accounted For = 0.99975.) See text for details.

The SNI distributions differed significantly among the 3 groups (P < 0.001 for each comparison, Kolmogorov-Smirnov test). The weighted MDS group configuration, based on Smirnov’s Z statistic as a distance measure between SNI distributions and 100 bootstrap proximity matrices (see Methods), is shown in Figure 10. It can be seen that the Control, GWI, and GWI + MH groups occupy 3 different quadrants of the 2-D MDS space, where the horizontal and vertical dimensions can be interpreted as relating to mental health status and presence of GWI, respectively. The occupied quadrants correspond to (a) the absence of GWI and mental health problems (Control group), (b) the presence of GWI but absence of mental health problems (GWI group), and (c) the presence of both GWI and mental health problems (GWI+MH group).

**Figure 11:**
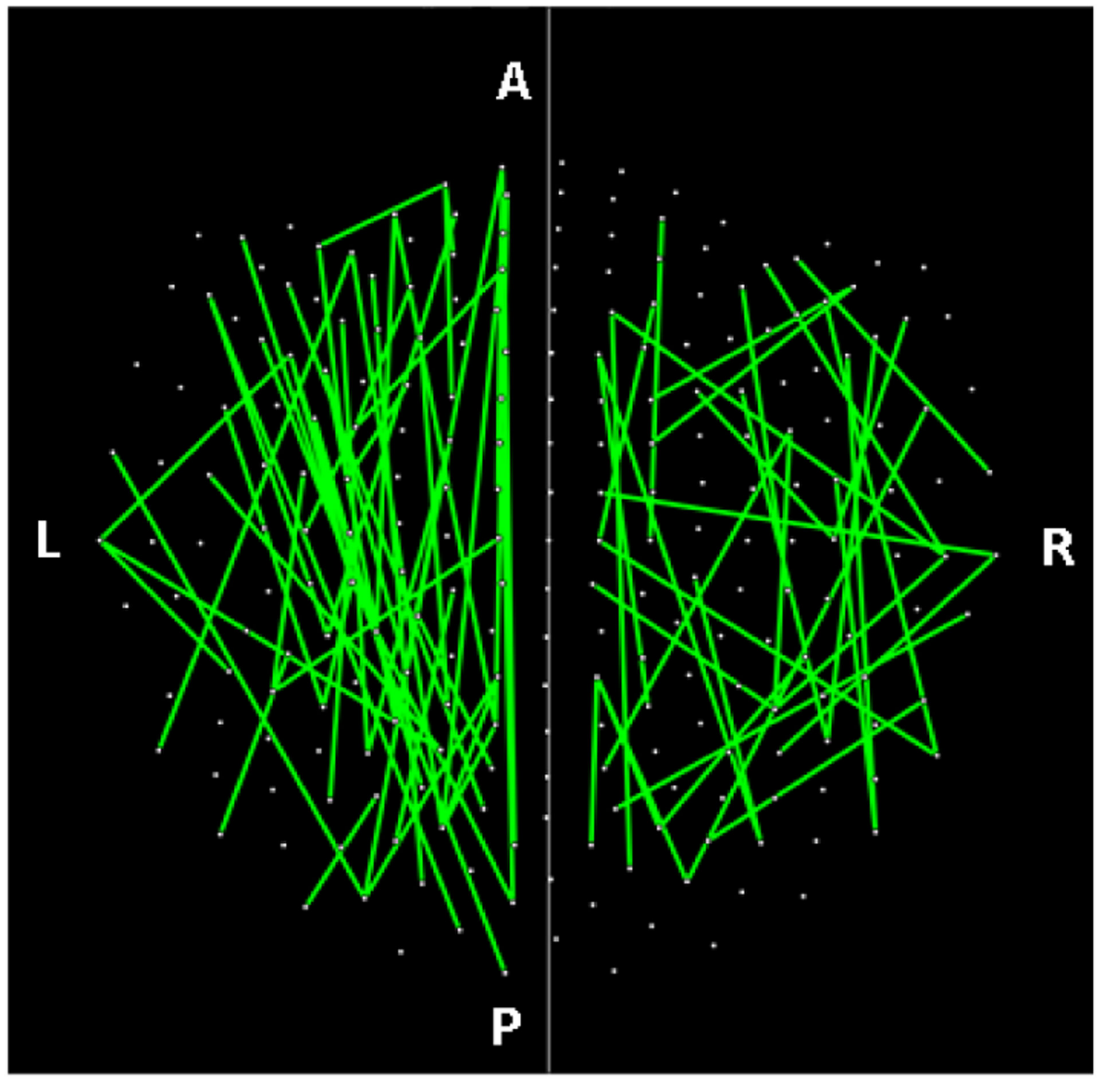
Lines indicate SNIs in the left and right hemisphere exceeding a nominal threshold of P < 0.01 (uncorrected) in the Control vs. GWI ANCOVA (F-test). There is a left hemispheric preponderance. A, anterior; P, posterior; L, left; R, right. (See text for details.)

**Figure 12:**
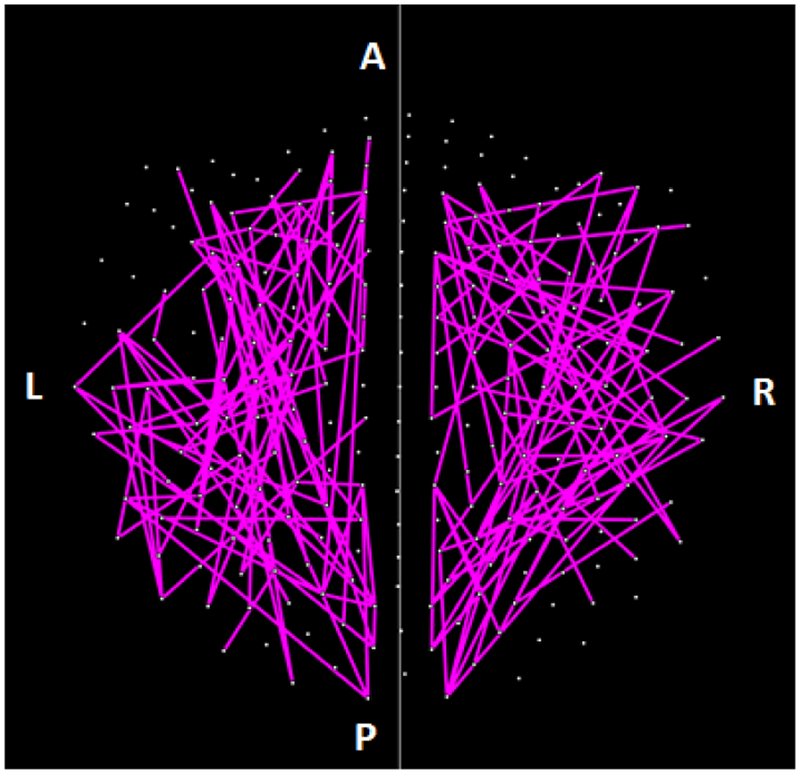
Lines indicate SNIs in the left and right hemisphere exceeding a nominal threshold of P < 0.01 (uncorrected) in the Control vs. GWI+MH ANCOVA (F-test). Compared to plot in Fig. 10, there is an increased left hemispheric involvement and a more prominent involvement of the right hemisphere. (See text for details.)

**Figure 13:**
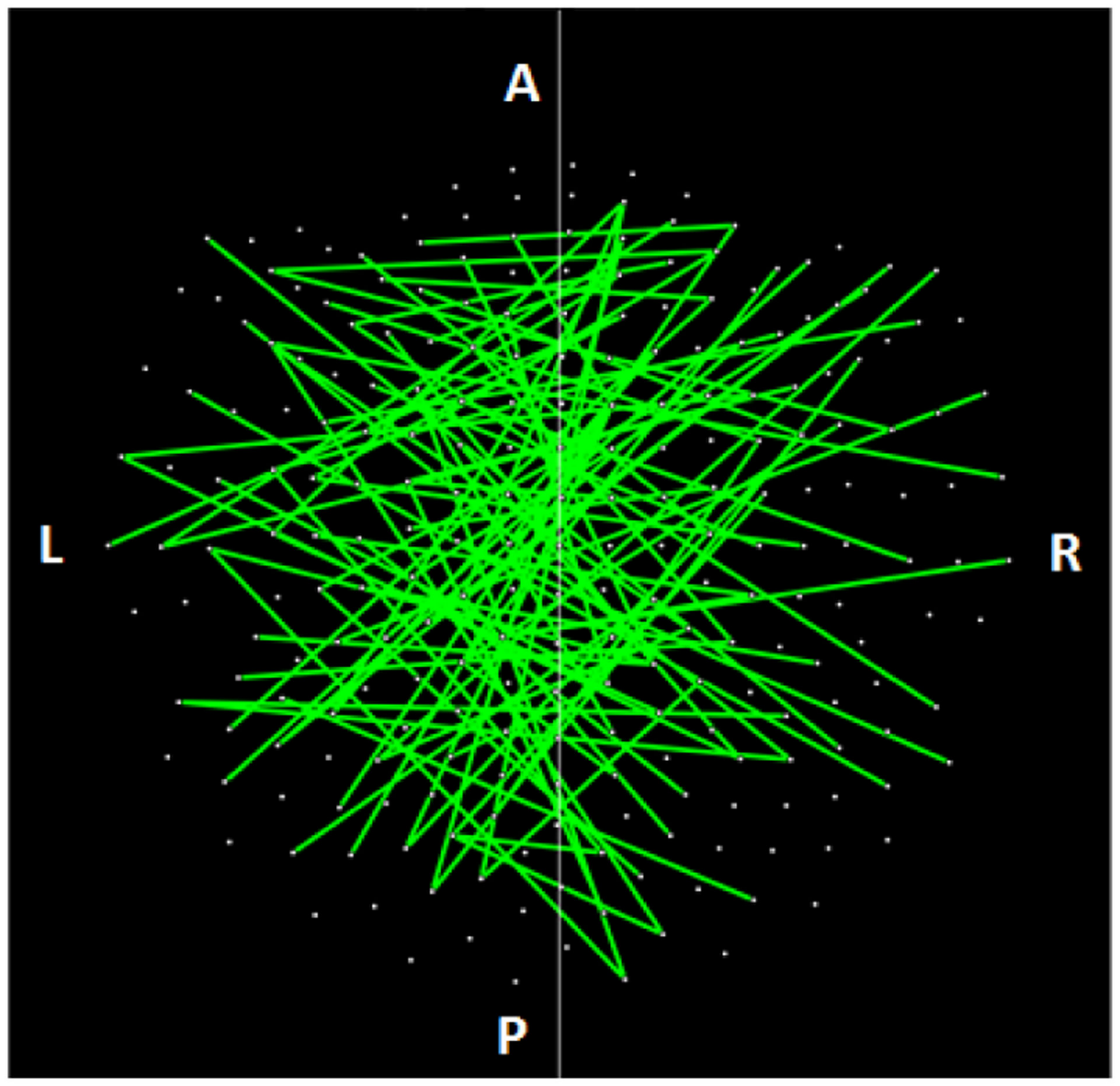
Lines indicate interhemispheric SNIs exceeding a nominal threshold of P < 0.01 (uncorrected) in the Control vs. GWI ANCOVA (F-test).

**Figure 14:**
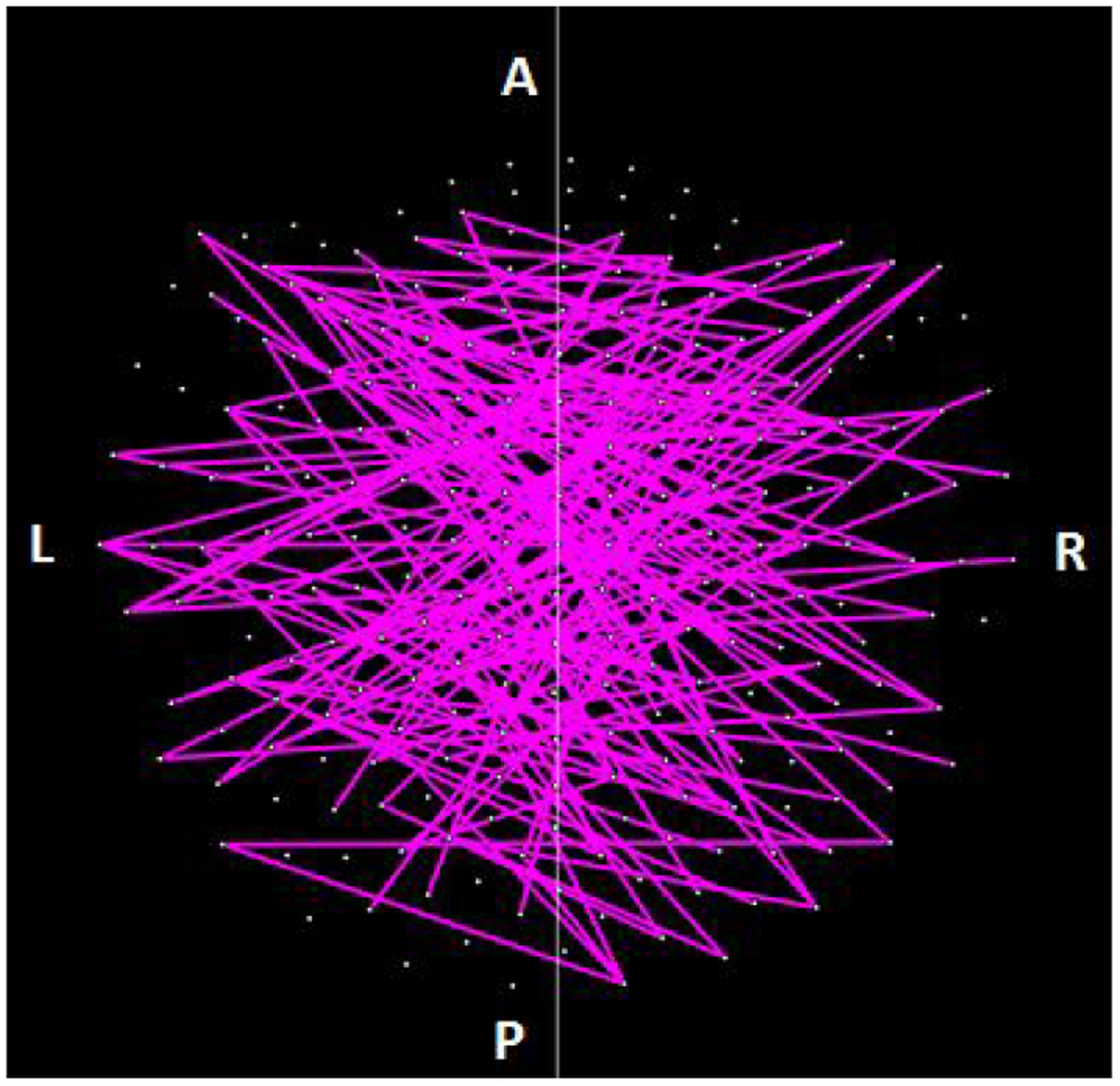
Lines indicate interhemispheric SNIs exceeding a nominal threshold of P < 0.01 (uncorrected) in the Control vs. GWI ANCOVA (F-test). Compared to plot in Fig. 12, there is an increased left hemispheric involvement and a more prominent involvement of the right hemisphere. (See text for details.)

The parametric ANCOVA carried out for each sensor pair yielded results that helped identify possible differential involvement of neural interactions within the left and right hemispheres, and between hemispheres, in group comparisons. First, for visualization purposes, we used a nominal threshold of P < 0.01 (uncorrected) in the Group F-test (ANCOVA) as a screening tool. The plots in Figures. 11–14 illustrate the distribution of these effects for the Control vs. GWI (within hemispheres, Fig. 11), Control vs. GWI+MH (within hemispheres, Fig. 12), Control vs. GWI (between hemispheres, Fig. 13), and Control vs. GWI+MH (between hemispheres, Fig. 14). It can be seen that GWI involved predominantly the left hemisphere (Fig. 11). This was enhanced in the presence of mental health disorders, together with a predominant involvement of the right hemisphere (Fig. 12). Interhemispheric interactions were more numerous in the Control vs. GWI+MH comparison (Fig. 14) than the Control vs. GWI contrast (Fig. 13). Very similar patterns of differences were also observed when other thresholds were used (e.g. P < 0.05 or P < 0.005). These findings suggested that general measures of SNI distributions in the left and right hemisphere, and between hemispheres, might be useful as neural variables in distinguishing groups. For that purpose, we used, for each participant, the mean, the standard deviation, and the ratio of the mean over its standard error of the SNI distributions of the left hemisphere, the right hemisphere and of the interhemispheric SNIs for a total of 9 neural measures.

**Figure 15:**
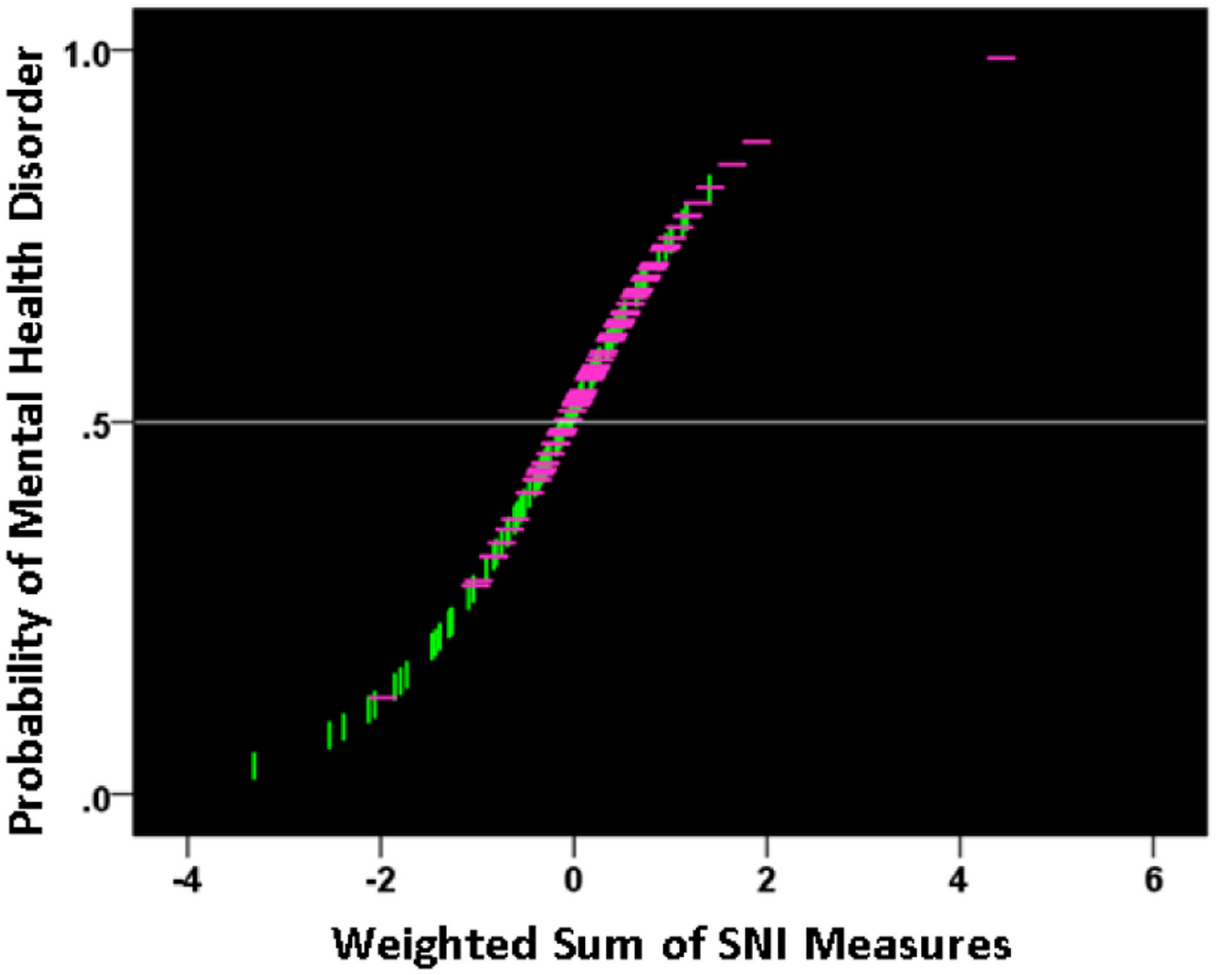
The probability of presence of mental health disorder is plotted against the weighted sum of the 9 SNI measures, where the weights are the coefficients provided by the logistic regression. (see text for details.) Vertical green lines indicate participants without mental health problems; horizontal magenta lines indicate participants with mental health problems.

**Figure 16:**
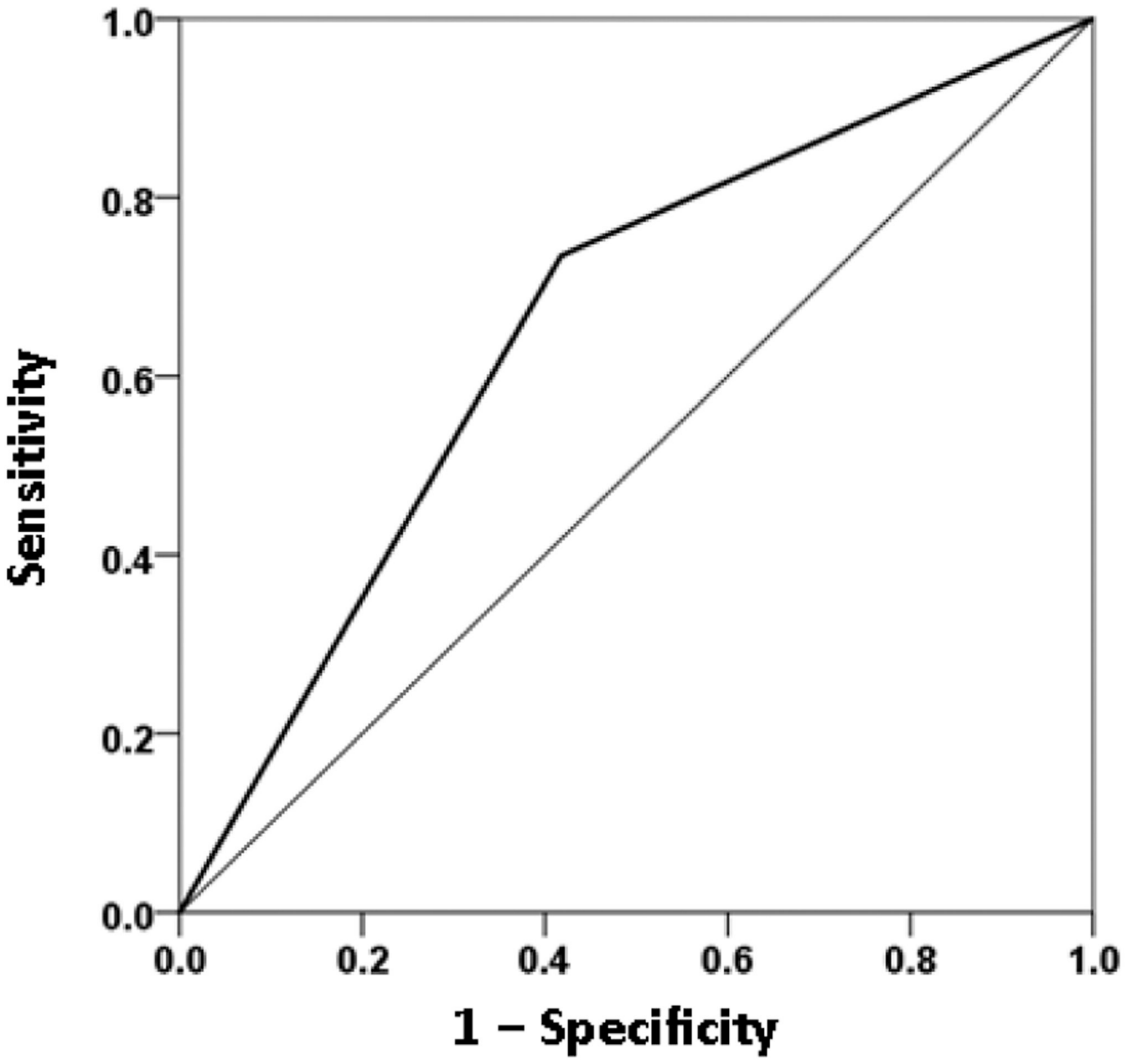
ROC curve yielded by the logistic regression of presence/absence of mental health disorder against the 9 SNI measures, adjusted for age and gender. (See text for details.)

We used these measures in a logistic regression analysis, analogous to the one we used above with GWI symptoms. For that purpose, we adjusted each neural measure for age and gender by performing a linear regression of each measure on age and gender, and then using the residuals as independent variables in the logistic regression analysis. All participants had valid SNI measures (total N = 91 GWI + 98 GWI+MH = 189). We found a significant dependence on the presence of mental health illness on these neural measures. Figure 15 plots the probability of a particular individual to have mental illness against the weighted sum of the 9 SNI variables. It can be seen that this probability increases as a sigmoid (logistic) function of the weighted linear sum of the neural measures. The Nagelkerke R^2^ was 0.156. The overall classification rate (with probability = 0.5 as the group discrimination border) was 66.1% (58.2% specificity, 73.5% sensitivity). The following statistics derived from the two-way table documented the high statistical significance of the obtained classification: χ^2^ = 19.5 (P = 0.00001, 2-sided); probability of Fisher’s exact test = 0.000016, 2-sided; odds ratio = 3.86 (asymptotic 2-sided significance P = 0.000015), lower bound of 95% confidence interval (CI) = 2.09, upper bound = 7.12). The receiver operating characteristic (ROC) curve (Figure 16) was 0.659, its asymptotic standard error 0.04, the asymptotic significance 0.00017, the lower bound of 95% CI 0.58 and the upper bound 0.73.

### Association of GWI symptom severity and synchronous neural interactions in the context of mental illness prediction

The results above show that the presence of a mental health disorder in veterans suffering from GWI can be reasonably well predicted by two separate, unrelated measures, namely GWI symptom severity and SNI distribution parameters. The weighted sums of these measures, based on the coefficients yielded by the logistic regression, form the horizontal axes in Figures 8 and 15, respectively. The question is whether these separate measures are associated, given that they both predict, independently, the presence of mental health disorder. For that purpose, we performed a correlation analysis between the two weighted sums above. The parametric Pearson correlation coefficient was 0.216 (P = 0.004, N = 174) and the nonparametric Spearman correlation coefficient was 0.302 (P = 0.00005, N = 174). (The higher value of the nonparametric correlation is due to the fact the distributions of the weighted GWI and SNI sums were slightly skewed, thus deviating from the normal distribution and resulting in loss of power.) This highly significant positive correlation between the weighted GWI and SNI measures provides a key link between GWI symptom severity and synchronous neural interactions *in the context of mental illness*.

## Discussion

In this study we investigated in detail two main questions concerning GWI, namely, first, its relations to mental health disorders and, second, its underlying neural mechanisms. Specifically, we tested the hypothesis that GWI is “nothing but PTSD” at the levels of both symptomatology and neurobiology. Our results demonstrated conclusively that GWI is a distinct illness that may exist independently of PTSD or other mental health disorders, with distinct symptomatology and neural mechanisms involving predominantly the left hemisphere.

### GWI symptom severity, structure, and relation to mental health problems

With few exceptions, GWI symptom severity increased systematically from controls to GWI to GWI+MH. Among those with both GWI+MH, fatigue, gastrointestinal problems, and neurocognitive problems were most enhanced relative to GWI alone. Notably, the internal structure of GWI symptoms did not differ between the GWI and GWI+MH groups, yet was remarkably different in the control group. Specifically, GWI symptoms were represented by a single common factor in the GWI groups and by 3 factors in the control group. The common structure across both GWI groups suggests that GWI is independent of mental health symptoms, a finding that was further substantiated by the lack of significant associations between the PTSD PCL score and GWI symptom domains. The lack of influence of mental health symptoms on GWI symptom domains stands in contrast to prior theories that GWI essentially reflected PTSD and related mental health problems. Our findings suggest just the opposite, namely that increased GWI symptom severity may, in fact, increase the likelihood of having a mental health disorder. Although the mechanisms underlying this interaction are not known, it is reasonable to suppose that GWI confers a sensitization to key neural cellular processes thus rendering the brain more vulnerable to traumatic insults which may lead to PTSD. Such sensitization may be due to lack of protective immune mechanisms^13^, as discussed below.

### Neural mechanisms

In a previous study^12^, we showed that GWI could be characterized by anomalies in synchronized neural activity. In keeping with this finding, here we found, additionally, that the GWI group was clearly discriminated from the Control and GWI + MH groups and that it occupied its own distinct space in the MDS plot. This finding again dispels the notion that GWI can be accounted for by mental disorder^6^ and provides clear objective evidence of altered neural functioning in GWI.

Further evaluation of the altered neural interactions within and across hemispheres demonstrated distinct patterns of neural activity across groups. Specifically, within hemisphere analyses revealed that GWI was characterized primarily by left hemispheric involvement relative to controls. The presence of mental health problems was associated with additional left hemispheric neural anomalies as well as substantially increased right hemispheric involvement. Interhemispheric analyses revealed markedly increased and more diffuse neural anomalies in the GWI+MH group (vs. controls) relative to the GWI group (vs. Controls). Notably, temporal areas were largely spared in the GWI vs. Control contrast yet were substantially involved in the GWI+MH vs. Control contrast. We have previously demonstrated the involvement of temporal anomalies in PTSD^9,10^. The present findings substantiate the role of temporal regions in PTSD and related mental health conditions, but not in GWI, again pointing to the distinction between GWI and PTSD and highlighting the discriminatory power of SNI^8,20^. Remarkably, properties of the SNI distributions within and across hemisphere were highly predictive of mental health status.

### GWI severity and comorbid diagnosable illnesses

We found that, independently, self-reported GWI symptoms and brain SNI could predict mental health status in veterans with GWI. Furthermore, these measures were highly significantly correlated. This relation – specifically, that GWI severity predicts mental health status – was unexpected although not entirely surprising. GWI is characterized by involvement of multiple systems including musculoskeletal, neural, respiratory, gastrointestinal, and dermal. Here, where the focus was on neural functioning, we found that as GWI symptoms increased, neural (i.e., SNI) anomalies increased, as did the probability of diagnosable mental health problems. Given the correspondence between mental health problems and both structural and functional neural anomalies, it follows that as the brain is increasingly affected by GWI, mental health symptoms may arise. As overall GWI severity increases, it is likely that multiple systems are affected in parallel. For instance, fatigue, gastrointestinal, and musculoskeletal symptoms may reach a certain threshold resulting in physician-diagnosed conditions such as chronic fatigue, irritable bowel syndrome, and fibromyalgia, respectively. Indeed, these diagnoses (and several others) are much more common in veterans with GWI, regardless of deployment status, relative to those without GWI^2,36^.

The involvement of multiple systems in GWI is comparable to the widespread effects of known autoimmune disorders such as systemic lupus erythematosus and multiple sclerosis, among others. We have shown recently that brain mechanisms are very similar between GWI and multiple sclerosis, Sjögren’s syndrome, and rheumatoid arthritis^15^. Furthermore, we have recently demonstrated a genetic susceptibility involving the human leukocyte antigen (HLA) class 2 genes^13^, which are central to specific immunity. Taken together, these findings suggest that GWI involves immune system dysfunction which may manifest as a disruption in various systems.

### Conclusions and future directions

**Figure 17:**
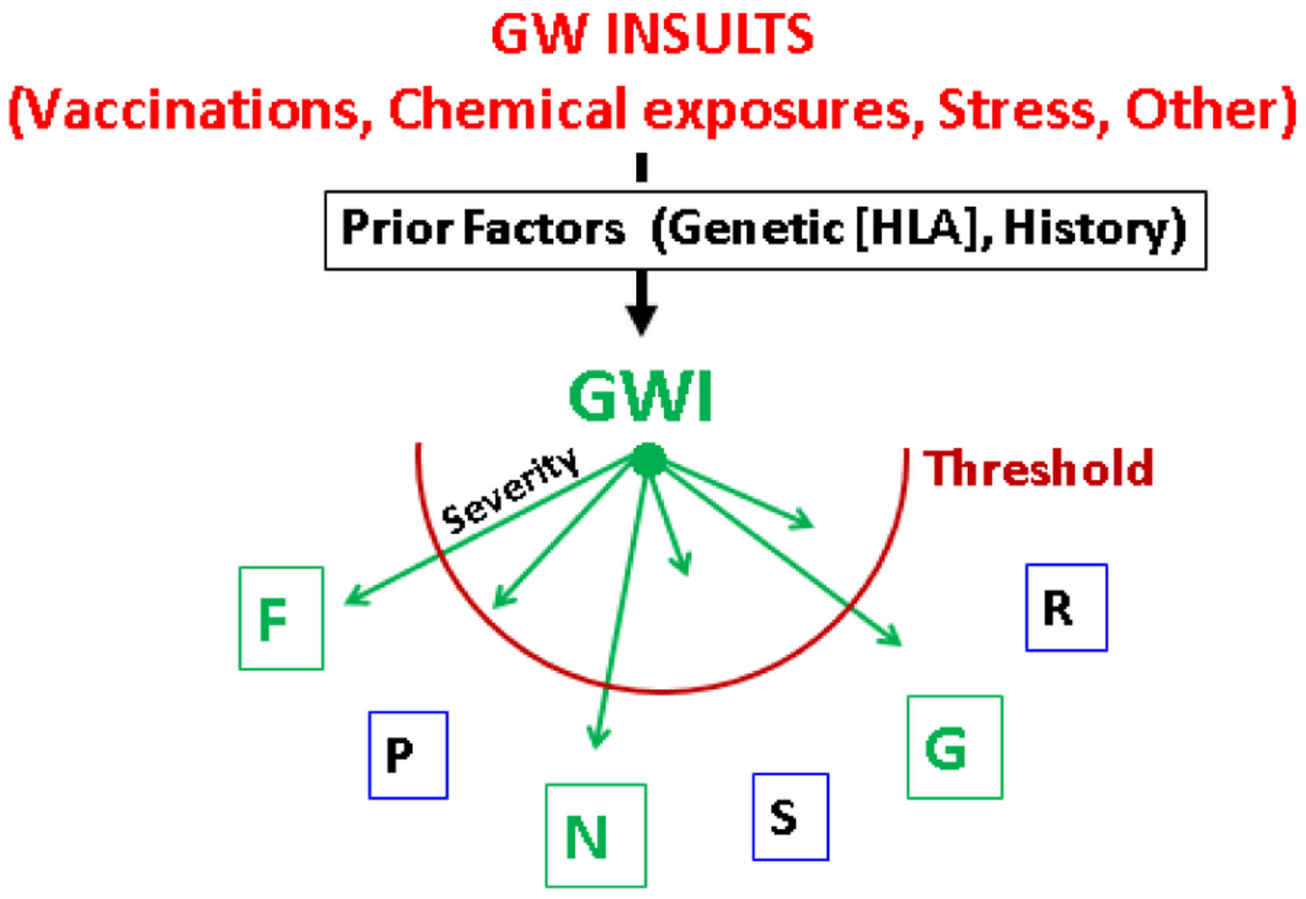
Proposed framework of the various factors involved in GWI. Abbreviations as in Fig. 5. Symptoms exceeding threshold (in green) indicate diagnosable disease.

Despite more than two decades of research, the pathophysiology of GWI remains poorly understood. Here, we have demonstrated altered brain synchronicity in GWI that predominantly affected the left hemisphere. This adds to the relatively small body of research that has demonstrated brain abnormalities associated with GWI and/or various deployment-related chemical exposures^37^. Additional research using other neuroimaging techniques (e.g., magnetic resonance imaging) has proved useful in further localizing brain areas that are affected in GWI and how those changes may, in turn, give rise to mental health symptoms. To that end, we have demonstrated the presence of subcortical brain atrophy in GWI^38^. The present study identified abnormalities in brain function associated with GWI and PTSD, and underscores the discriminatory power of SNI. The potential clinical implications appear clear: this line of investigation can lead to improvements in differential diagnosis and therefore improve treatment for veterans suffering from GWI. To that end, a more general understanding of the interplay of various factors involved in GWI would be helpful, as outlined in our proposed framework illustrated in Figure 17.

As mentioned above, another line of research concerns the interplay of neural functioning and genetic factors in GWI. We recently demonstrated protective effects of certain HLA genes such that GWI symptom severity was inversely associated with the number of copies of 6 alleles that successfully discriminate GWI vs. Control participants^13^. With respect to brain structure, we demonstrated that subcortical brain atrophy in GWI^38^ is prevented by HLA DRB1*13:02 allele^39^, one of the 6 HLA class 2 alleles we identified previously as protective for GWI^13^. Finally, relating that to neural functioning, we would expect the discriminatory HLA alleles to exert protective effects on SNI such that individuals with more discriminatory allele copies would exhibit fewer aberrant SNI effects and reduced symptom severity in domains directly related to neural function (e.g., neurocognitive/mood symptoms). Indeed, we found that SNI abnormalities in GWI are observed mostly in brain territories unrelated to the 6 HLA protective alleles^15^. Interestingly, changes in expression of intercellular cell adhesion molecule 5 (ICAM-5) in PTSD have been reported recently^40^, a molecule that is expressed only in the brain where it modulates synapse formation, immune function and inflammation^41^. Thus, regulation of immune function could be a confluent substrate for the GWI-PTSD association described in this study. An investigation of ICAM-5 expression (or concentration) in GWI remains to be carried out.

## Figures and Tables

**Table 1.** Demographic characteristics of study participants.

	Control	GWI	GWI+MH
Age			
Mean	52.4	50.7	48.9
SD	10.9	8.2	7.7
N	41	91	98
Gender			
Male	40	80	86
Female	1	11	12

**Table 2.** Severity of individual symptoms as percentage of total severity.

	Control	GWI	GWI+MH
Fatigue	14.3	28.1	27.5
Pain	37.4	27.1	23.9
Neurocognitive	12.5	14.0	16.3
Skin	14.0	14.0	8.5
Gastrointestinal	20.2	8.7	14.2
Respiratory	1.6	8.1	9.6
	100	100	100%
